# Prefecture-Level Association Between Reported Syphilis Cases and Sexually Oriented Establishments in Japan: An Ecological Study

**DOI:** 10.7759/cureus.104204

**Published:** 2026-02-24

**Authors:** Daiji Takamoto, Takashi Kawahara, Karibe Jurii, Kuroda Shinnosuke, Jun-ichi Teranishi, Kazuhide Makiyama

**Affiliations:** 1 Urology and Renal Transplantation, Yokohama City University Medical Center, Yokohama, JPN; 2 Urology, Yokosuka Kyosai Hospital, Yokosuka, JPN; 3 Urology, Yokohama City University, Yokohama, JPN

**Keywords:** ecological study, japan, public health, sex industry, sexually transmitted infections, syphilis

## Abstract

Background and aim

The number of reported syphilis cases has increased in Japan in recent years, while the contribution of structural and regional factors remains unclear. Japan maintains a distinctive legal and operational framework for the sex industry, characterized by geographic concentration of establishments and expansion of non-storefront services. This study aimed to examine the prefecture-level association between reported syphilis cases and the number of sexually oriented establishments in Japan.

Methods

This ecological study analyzed publicly available prefectural data on reported syphilis cases from April 2023 to March 2024, obtained from the national infectious disease surveillance system. Data on the number of sexually oriented establishments were derived from statistics published by the Japan National Crime Prevention Association. For comparison, prefecture-level data on chlamydia and gonorrhea were also analyzed. Infection rates were calculated per 1,000,000 population. Simple linear regression analyses were conducted to evaluate associations between infection rates and the number of establishments.

Results

A significant positive association was observed between the number of sexually oriented establishments and the reported incidence of syphilis (β=0.005535, R²=0.5157, p<0.0001). No statistically significant associations were identified for chlamydia (p=0.8485) or gonorrhea (p=0.3457).

Conclusions

At the prefectural level in Japan, the number of sexually oriented establishments was significantly associated with reported syphilis incidence, whereas no such association was observed for chlamydia or gonorrhea. Although causality cannot be inferred from this ecological analysis, these findings suggest that regional structural factors may be associated with syphilis transmission dynamics. Public health strategies in high-burden regions may benefit from continued emphasis on education, testing, and timely treatment.

## Introduction

Syphilis remains a significant public health concern worldwide, and the number of reported cases has increased in several developed countries over the past decade [1,2]. In Japan, reported syphilis cases have shown a marked and sustained increase since around 2015, prompting concerns regarding changes in sexual behavior, population mobility, and the effectiveness of current prevention strategies [3]. Despite extensive epidemiological surveillance, the contribution of regional and structural factors to this increase has not been fully elucidated.

Japan has a distinctive legal and operational framework governing the sex industry. Although prostitution has been prohibited since the enactment of the Prostitution Prevention Act (1956; fully enforced 1958), various forms of sexually oriented services continue to operate legally under the Adult Entertainment Business Law [4,5]. In contrast to many other countries, the establishment of new storefront businesses is highly restricted, while existing businesses are permitted to continue operating in historically designated areas. As a result, sexually oriented establishments tend to be geographically concentrated, whereas non-storefront, dispatch-based services have expanded alongside urbanization and population growth [4,5]. These unique structural characteristics may influence regional patterns of sexually transmitted infection transmission.

Previous studies have examined behavioral and demographic factors associated with syphilis transmission; however, few have focused on the relationship between the distribution of sexually oriented establishments and syphilis incidence at a regional level, particularly in settings with a regulatory framework such as that of Japan [6]. Moreover, comparisons with other sexually transmitted infections, such as chlamydia and gonorrhea, which differ in incubation period and surveillance systems, may provide additional insights into disease-specific transmission dynamics [2,7].

Therefore, this study aimed to examine the prefecture-level association between reported syphilis cases and the number of sexually oriented establishments in Japan using publicly available surveillance data. In addition, we explored whether similar associations were observed for chlamydia and gonorrhea to better contextualize the findings related to syphilis.

## Materials and methods

Study design and data sources

This ecological study used publicly available prefecture-level surveillance data in Japan. Data on reported syphilis cases were obtained from annual infectious disease surveillance reports published by the National Institute of Infectious Diseases (NIID) [3]. Syphilis is classified as a category 5 notifiable infectious disease in Japan, and all confirmed cases must be reported within seven days of diagnosis. The study period spanned one year, from April 2023 to March 2024.

Data on sexually oriented establishments were obtained from publicly available statistics released by the National Police Agency and the Japan National Crime Prevention Association under the Adult Entertainment Business Law [4,5]. These data included both storefront and non-storefront establishments, dispatch-based sexually oriented businesses. Establishment data were derived from the 2024 fiscal year database, corresponding to the infectious disease surveillance period analyzed in this study.

Inclusion and exclusion criteria

All prefectures in Japan were included in the analysis. No individual-level patient data were used, and no exclusion criteria were applied, as all analyses were conducted at the prefectural level using aggregated data.

Comparison with other sexually transmitted infections

For comparative purposes, prefecture-level data on chlamydia and gonorrhea were obtained from national sentinel surveillance reports published by the Ministry of Health, Labour and Welfare. These infections are reported through a fixed-point surveillance system involving approximately 1,000 designated medical institutions nationwide. Sentinel site distribution may vary across prefectures, and differences in reporting coverage were not adjusted for in the present analysis.

Statistical analysis

Simple linear regression models were constructed with prefecture-level sexually transmitted infection (STI) incidence (per 1,000,000 population) as the dependent variable and the number of sexually oriented establishments as the independent variable. The number of sexually oriented establishments was analyzed as an absolute count per prefecture. We did not standardize establishment numbers per population, as our objective was to examine geographic structural concentration rather than per capita availability. However, population-adjusted establishment density may provide additional insight and should be examined in future studies.

Incidence rates were standardized as cases per 1,000,000 population using official prefectural population statistics published by the Statistics Bureau of Japan. Model assumptions, including linearity and residual distribution, were visually inspected using diagnostic plots. No extreme outliers requiring exclusion were identified. Statistical analyses were conducted using Prism version 9.5.1 (San Diego, CA: GraphPad Software). A two-sided p<0.05 was considered statistically significant.

## Results

The number of sexually oriented establishments and the reported incidence of syphilis, chlamydia, and gonorrhea in each prefecture are summarized in Table 1. Incidence rates were calculated as the number of reported cases per 1,000,000 population.

**Table 1:** Syphilis, chlamydia, and gonorrhea cases in Japan. *Values are expressed as the number of cases per 1,000,000 population. Prefecture-level distribution of reported syphilis, chlamydia, and gonorrhea cases in Japan. The number of reported cases for each infection was standardized as cases per 1,000,000 population using prefectural population statistics. Data were obtained from publicly available national surveillance reports for the period from April 2023 to March 2024.

Region	Prefectures	Number of sex industry establishments	Syphilis*	Chlamydia*	Gonorrhea*
Hokkaido	Hokkaido	1,156	11.06	40.81	11.65
Tohoku	Aomori	185	1.97	34.48	4.91
	Iwate	196	3.76	20.23	5.1
	Miyagi	380	6.77	25.63	7.47
	Akita	62	3.6	18.84	4.13
	Yamagata	162	3.13	14.69	3.51
	Fukushima	492	7.56	36.15	13.08
Kanto	Tokyo	3,947	25.3	16.14	7.43
	Ibaraki	733	11.15	34.15	7.68
	Tochigi	372	9.47	24.1	10.52
	Gunma	445	9.29	37.83	11
	Saitama	728	6.1	21.98	4.99
	Chiba	858	7.55	33.23	9.56
	Kanagawa	850	7.27	18.03	6.84
Chubu	Niigata	327	5.93	15.11	4.82
	Yamanashi	141	4.22	24.22	8.2
	Nagano	345	4.28	10.62	1.43
	Shizuoka	705	8.98	16.69	3.77
	Toyama	84	2.34	10.73	2.63
	Ishikawa	122	4.98	55.2	13.42
	Fukui	58	7.63	5.39	3.16
	Gifu	362	7.45	13.82	2.96
	Aichi	1,216	10.6	28.58	12.68
Kinki	Mie	224	5.69	11.79	5.3
	Shiga	173	5.03	3.19	0.85
	Kyoto	207	5.74	13.24	2.11
	Osaka	1,987	21.91	26.18	10.07
	Hyogo	490	8.93	21.58	5.82
	Nara	148	6.77	18.78	4.49
	Wakayama	83	6.89	24.84	10.72
Chugoku	Tottori	85	5.28	45.9	15.85
	Shimane	54	3.31	20.3	7.97
	Okayama	555	18.6	20.9	5.28
	Hiroshima	570	14.03	31.01	12.66
	Yamaguchi	243	7.08	28.69	10.02
Shikoku	Tokushima	198	10.11	36.52	8.43
	Kagawa	335	14.33	13.59	4.25
	Ehime	175	11.05	14.99	8.1
	Kochi	128	7.6	6.87	1.61
Kyushu	Fukuoka	1,456	18.38	27.22	9.33
	Saga	76	10.79	36.35	10.17
	Nagasaki	101	11.1	18.12	6.55
	Kumamoto	335	14.12	50	16.61
	Oita	440	6.82	16.34	6.19
	Miyazaki	347	16.02	24.41	9.71
	Kagoshima	457	10.85	41.24	21
	Okinawa	261	8.86	26.23	4.56

**Figure 1:**
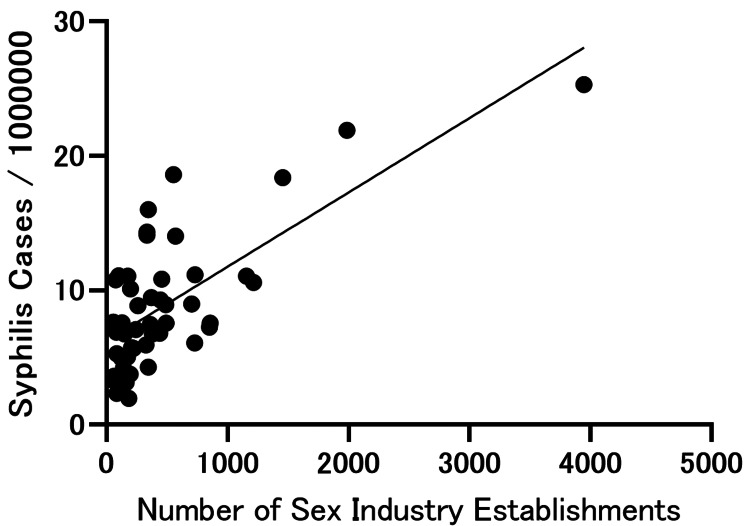
Association between the reported incidence of syphilis and the number of sexually oriented establishments across prefectures in Japan. Each point represents a prefecture. Syphilis incidence is expressed as cases per 1,000,000 population. The line indicates the simple linear regression fit.

**Figure 2:**
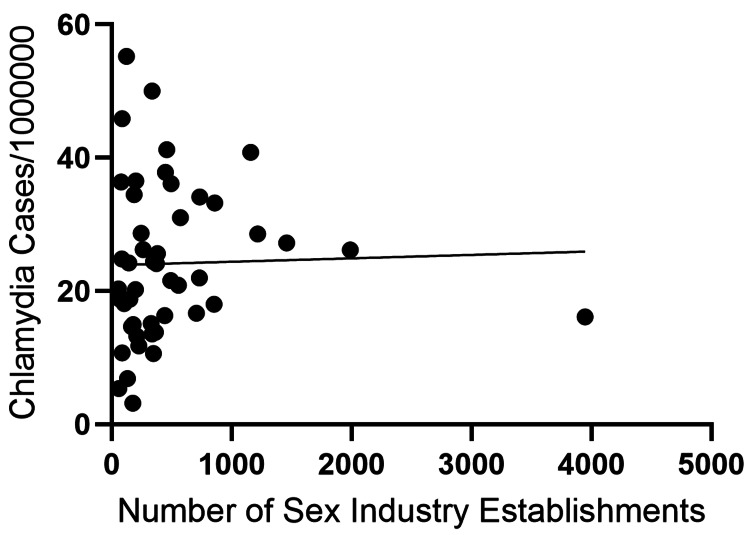
Association between the reported incidence of chlamydia and the number of sexually oriented establishments across prefectures in Japan. Each point represents a prefecture. Chlamydia incidence is expressed as cases per 1,000,000 population based on sentinel surveillance data. The line indicates the simple linear regression fit.

**Figure 3:**
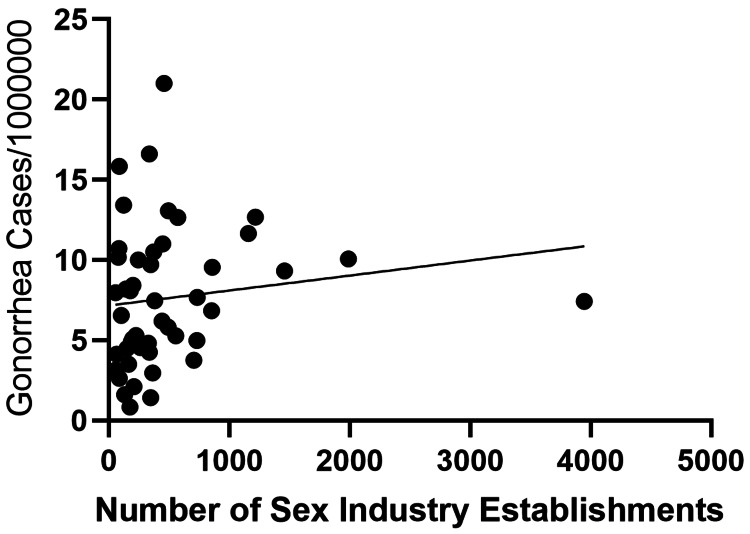
Association between the reported incidence of gonorrhea and the number of sexually oriented establishments across prefectures in Japan. Each point represents a prefecture. Gonorrhea incidence is expressed as cases per 1,000,000 population based on sentinel surveillance data. The line indicates the simple linear regression fit.

Simple linear regression demonstrated a significant positive association between the number of sexually oriented establishments and the reported incidence of syphilis (β=0.005535, R²=0.5157, p<0.0001) (Figure 1). In contrast, no statistically significant associations were observed for chlamydia (β=0.0005145, R²=0.0008202, p=0.8485) or gonorrhea (β=0.0009341, R²=0.01978, p=0.3457) (Figures 2, 3).

## Discussion

In this ecological study, we identified a significant prefecture-level association between the reported incidence of syphilis and the number of sexually oriented establishments in Japan (β=0.005535, R²=0.5157, p<0.0001). In contrast, no statistically significant associations were observed for chlamydia (p=0.8485) or gonorrhea (p=0.3457). These findings suggest that structural and epidemiological factors contributing to syphilis transmission may differ from those of other common sexually transmitted infections; however, interpretation requires caution given the ecological design and surveillance differences.

Syphilis has been reported in Japan for centuries, and historically, its transmission was frequently linked to commercial sexual contact and subsequent spread to the broader population. In recent decades, the epidemiology of syphilis has become increasingly complex owing to diversification of sexual practices, increased population mobility, and changes in social behavior [1,2]. Consistent with national surveillance data, Japan has experienced a sustained increase in reported syphilis cases since the mid-2010s, underscoring the importance of understanding contextual and structural determinants of transmission [3].

Japan maintains a distinctive regulatory framework governing the sex industry. Although prostitution has been prohibited since the enactment of the Prostitution Prevention Act (1956; fully enforced 1958), various forms of sexually oriented services continue to operate legally under the Adult Entertainment Business Law [4,5]. The establishment of new storefront businesses is highly restricted, whereas existing establishments are permitted to continue operating in historically designated areas. Consequently, sexually oriented establishments are geographically concentrated, while non-storefront, dispatch-based services have expanded alongside urbanization and population growth. These structural characteristics may influence regional transmission dynamics by shaping patterns of sexual contact networks.

Several hypotheses may explain why an association was observed for syphilis but not for chlamydia or gonorrhea. Syphilis has a relatively long incubation period, and early manifestations may be mild or unrecognized, potentially allowing repeated sexual contact prior to diagnosis and treatment [8]. In contrast, chlamydia and gonorrhea are often detected earlier through symptoms or screening programs, prompting treatment that may reduce onward transmission [9,10]. In addition, prior network analyses have demonstrated that commercial sex environments frequently involve repeat clients and clustered sexual contact structures, which may facilitate sustained transmission of infections with longer incubation periods [11]. However, these mechanistic interpretations remain speculative and cannot be confirmed within the constraints of an ecological analysis.

Importantly, differences in surveillance systems substantially limit comparability across infections. Syphilis is subject to mandatory full-case reporting in Japan, whereas chlamydia and gonorrhea are monitored through sentinel surveillance systems involving approximately 1,000 designated medical institutions nationwide [3,7]. Sentinel site density, geographic distribution, and healthcare-seeking behavior may vary across prefectures, potentially resulting in differential under-ascertainment. Therefore, the absence of association for chlamydia and gonorrhea may reflect surveillance structure and measurement bias rather than true epidemiological differences. The negative findings for these infections should thus be interpreted cautiously, and direct comparisons between infections may be methodologically constrained.

Our findings are partially consistent with prior studies examining social and behavioral correlates of syphilis transmission in Japan. For example, associations between syphilis incidence and dating application use have been reported, suggesting that evolving sexual networking patterns contribute to the epidemic [6]. The present study extends this literature by demonstrating that prefectural variation in the distribution of sexually oriented establishments is also correlated with syphilis incidence. However, this association does not imply causality and should be considered hypothesis-generating.

This study has several limitations. First, as a cross-sectional ecological analysis, it cannot establish temporal relationships or individual-level causation. Second, the analysis did not adjust for potential confounders such as population density, urbanization, healthcare access, tourism, or testing practices. Third, sexually oriented establishment counts were analyzed as absolute numbers rather than per capita measures, which may reflect broader population distribution patterns. Fourth, differences in surveillance systems introduce potential measurement bias. Finally, no data were available regarding establishment clientele characteristics, individual risk behaviors, or contact tracing information. Although Tokyo had the highest number of establishments and incidence rates, visual inspection of regression diagnostics suggested that the observed association was not solely driven by a single influential prefecture.

Despite these limitations, this study has several strengths, including nationwide coverage across all 47 prefectures, use of mandatory reporting data on syphilis, transparent, publicly available data sources, and inclusion of comparator infections to assess the specificity of the findings. Collectively, these features enhance reproducibility and contextual interpretation.

Overall, within Japan’s unique regulatory and structural context of the sex industry, we observed a significant prefecture-level association between the number of sexually oriented establishments and reported syphilis incidence. However, given the ecological design and surveillance heterogeneity, these findings should be interpreted as hypothesis-generating rather than causal evidence. Future studies incorporating longitudinal data and adjustment for population and behavioral confounders are warranted to clarify the role of structural factors in syphilis transmission dynamics.

## Conclusions

Within Japan’s distinctive regulatory context, we observed a significant prefecture-level association between the number of sexually oriented establishments and reported syphilis incidence, whereas no such association was identified for chlamydia or gonorrhea. Given the ecological design and surveillance heterogeneity, causal inference cannot be made. These findings should be considered hypothesis-generating and highlight the importance of future longitudinal studies incorporating population-adjusted exposure measures and surveillance intensity adjustments.
